# Cancer patients' preferences for written prognostic information provided outside the clinical context

**DOI:** 10.1038/sj.bjc.6601287

**Published:** 2003-10-14

**Authors:** H M Davey, P N Butow, B K Armstrong

**Affiliations:** 1 School of Public Health, Edward Ford Building – A27, The University of Sydney NSW 2006, Australia; 2 Department of Psychological Medicine, Blackburn Building D06, The University of Sydney NSW 2006, Australia

**Keywords:** prognosis, written information, patient preferences, understanding

## Abstract

Cancer patients' preferences for written prognostic information independent of the clinical context have not previously been investigated. This study aimed to assist a state cancer organisation to provide information to patients by assessing patients' understanding of statistical information; eliciting their preferences for framing, content and presentation; and assessing the acceptability of a card sort for obtaining preferences. With the exception of *conditional* and *relative* survival, initial difficulties in understanding statistical concepts were improved with a plain language explanation. Analysis of the interview transcripts revealed that participants generally supported the provision of written information about survival in booklets and on the Internet. They wanted positive, relevant and clear information. Participants said that the use of, and preferences for, this information would be affected by a patient's age, time since diagnosis, ability to cope with having cancer and the perceived credibility of the information source. They found the card sort acceptable, saying it made the assessment of understanding and selection of preferences easy. This study has identified two fundamental, and sometimes conflicting, factors underlying patients' preferences: the communication of hope and the need to understand information it has also identified patient characteristics thought to influence preferences. These factors and characteristics need to be taken into account when developing written prognostic information for patients.

More patients are told their diagnosis than prognosis (Charlton, 1992; Butow *et al*, 1996). Reticence to provide prognostic information may be due to concerns that patients misunderstand it (Mackillop *et al*, 1989; Haidet *et al*, 1998; Weeks *et al*, 1998), that it is contrary to their wishes or best interests (Christakis, 1999; Kaplowitz *et al*, 1999) or to the complexity of prognostic information. In addition to relevant figures differing according to diagnosis, staging and individual patient characteristics, translating group statistics into something that has meaning for the individual is difficult for both the clinician and the patient.

Moreover, considerable debate surrounds the best methods for disclosing prognostic information as patients vary in their preferences for the framing and presentation of information. In a questionnaire study of 100 women with breast cancer, Lobb *et al* (2001) asked women to select their preferred frame from a positive and a negative statement and a no preference option. Positively framed information was preferred by 43%, negatively framed by 33% and the remainder had no preference. They also found that women prefer prognostic information presented as words rather than numbers. However, the survey assessed preferences for only two presentation formats, when it is possible to present information in a greater range of ways (Lipkus and Hollands, 1999).

Although most cancer patients want prognostic information in a clinical setting (Jenkins, 2001), we found no published study that assessed whether patients want written prognostic information independent of the clinical setting. Similarly, it is unclear to what extent preferences for this written information would mirror those for the verbal communication of prognosis in the clinical setting. Within this setting, patients want doctors to ask if they want prognostic information; make sure someone is with them when it is given; summarise, write down and provide an audiotape of what they (the doctors) have said; explain any medical terms and check that they (the patients) understand; emphasise the good aspects; provide an opportunity to ask questions and to clarify the information; and ask if they want a second opinion (Lobb *et al*, 1998).

This study was carried out to provide guidance to The Cancer Council New South Wales on how it might present written prognostic information directly to the public. It aimed to obtain patients' views on the provision of statistical information about survival from cancer in a written form (booklet and Internet); elicit patients' preferences for the framing, content, presentation and differentiation by stage of this information; and determine the acceptability of a card sort method for eliciting patients' understanding of statistical terminology and preferences.

## MATERIALS AND METHODS

### Participants

Participants were cancer patients aged 18 years or older whose oncologists said were capable of completing a questionnaire and face-to-face interview in English.

### Materials

#### Questionnaire

The self-administered questionnaire elicited information on participants' age, gender, marital status, highest level of education, current or last employment occupation, diagnosis, time since diagnosis and treatment received.

#### Interview

The in-depth, face-to-face interview used semistructured questions and detailed prompts. A scenario was developed of women aged 75–89 years with cancer ‘x’ to prevent the naming of an actual cancer causing relevance bias. Statistics for this scenario were presented on cards to elicit participants' understanding and preferences. To ensure that the statistics were plausible, they were based on colorectal cancer, a common cancer with a relatively good survival in the early stages (Tracey and Supramaniam, 2002). All statistics were derived from The New South Wales Central Cancer Registry. Participants were asked to select their preferences based on what they wanted to see in a booklet or on the Internet. All cards were presented in random order. Unless otherwise stated, all information was provided for ‘*early stage cancer x*’ only.

Five issues were explored using the cards: understanding and preferences for framing, content, presentation and differentiation by stage.

**Figure 1:**
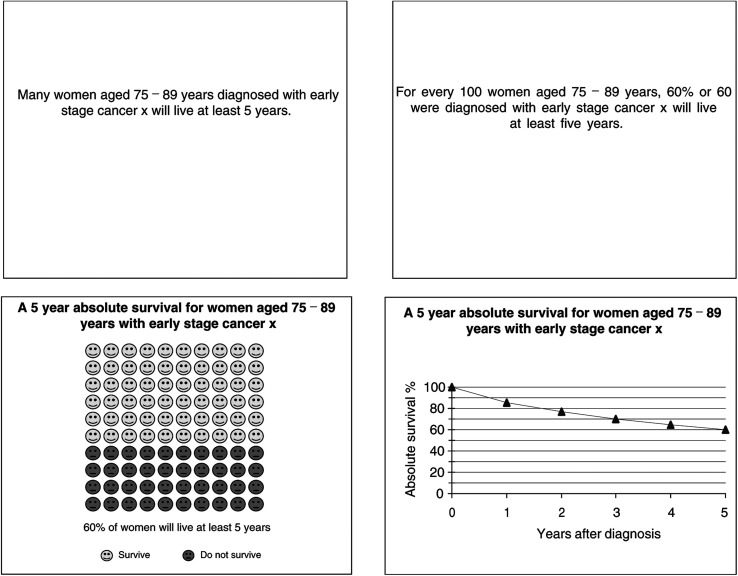
An example of the four presentation formats.

**Figure 2:**
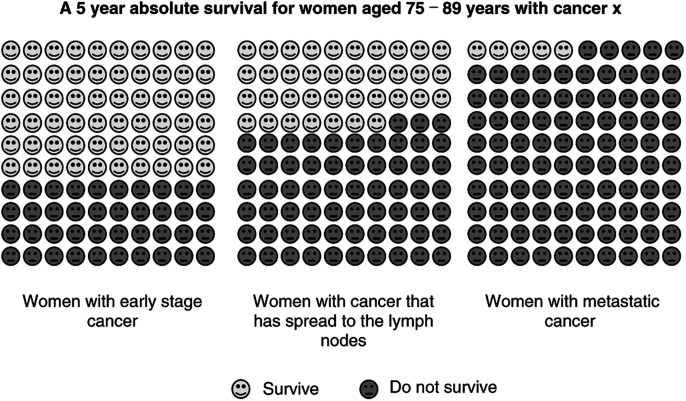
An example of a card used to elicit participants' stage differentiation preference.

1. *Understanding*: Participants were shown seven cards, each addressing one of *median survival*, *remaining lifespan*, *absolute survival*, *relative survival*, *conditional survival and a graph* (two cards). Each card contained a statement (e.g. ‘*The median survival time for early Cancer X is 6 years*.’) and four multiple choice response options (one correct response, two incorrect responses and one response stating ‘*I don't understand [the concept]*’). Participants were asked to select the most correct option. At the end of this section, any misunderstood concepts were explained.
2. *Framing preference:* Participants were shown three cards: ‘50% of women aged 75–89 years with early stage cancer x will live at least 6 years^1^ (*positive frame*), 50% of women aged 75–89 years with early stage cancer x will die within 6 years^1^ (*negative frame*) and a *mixed frame* card showing both statements. They were asked which frame they liked most; which they liked least; their reasons for this; whether the way information is expressed is important; and the emotional and other effects of framing. All subsequent preferences were elicited using the participant's preferred frame.
3. *Content preference*: Participants were shown nine different cards, each addressing one of *median survival*, *conditional median survival*, *change to life expectancy*, *5-year absolute survival*, *10-year absolute survival*, *5-year relative survival*, *10-year relative survival*, *conditional 5-year absolute survival and conditional 5-year relative survival* (the last two cases applied to women who had already survived 5 years). To minimise any effect of understanding and presentation style, all information was written in plain language using numbers (out of 100) and percentages. Participants were asked which two cards they most preferred; which two they least preferred; and their reasons for these preferences, including the clarity, importance, usefulness and emotional impact of the information.
4. *Presentation preference*: Participants were shown four presentation formats (100 faces, numbers and percentages, text only, and graph; see Figure 1

) for each of the two content cards they most preferred. They were told that each card contained the same information presented in a different way. They were asked to select their most and least preferred presentation formats and the reasons for these preferences.
5. *Stage differentiation preference*: The Cancer Council can produce information for three stages of cancer – local, regional and distant. Each participant's two most preferred content cards for early-stage cancer were compared to identical information for all stages (for example, see Figure 2

) and asked if they preferred one stage alone or three stages together and the reasons for this preference.

### Procedure

A qualitative research method was used because the research subject has been little studied (Miles and Huberman, 1994). Two medical oncologists recruited patients, one through his private rooms in a regional centre and the other through a public cancer clinic in a large city. Thus both urban and semirural patients' views were represented. The oncologists screened consecutive patients for eligibility and gave a brief explanation of the study. Interested patients received an information package and were asked to return the consent form to the first author (HD), who then arranged an interview. Slow initial recruitment prompted a variation on this procedure: eligible patients were identified from the clinic oncologist's lists; he sent them a letter introducing the study. Recruitment then proceeded as above.

HD conducted all interviews at venues nominated by participants. Participants completed the questionnaire followed by the interview. Interviews were tape recorded with the participants' permission. Using a grounded theory approach (Glaser and Strauss, 1967), new concepts that emerged in an interview were explored in subsequent interviews. All interviews were transcribed.

HD and PB independently reviewed and then discussed the first five tapes. A minor amendment was made to the interview with HD checking participants' understanding of statistical concepts that they originally misunderstood before eliciting their content preferences. After 26 interviews, a further review showed that information redundancy had been reached in the last three interviews and data collection ceased.

### Analysis

Frequency counts were carried out using SPSS (1999) v10.0. for Windows. HD analysed the transcripts using the constant comparative method (Glaser and Strauss, 1967), which involves identifying participants responses and comparing and contrasting them to identify recurring ideas and themes. Once completed, HD and PB met to review the analysis. Any differences in opinion were resolved by discussion.

The Human Research Ethics Committees of The Cancer Council, The Central Sydney Area Health Service and The University of Sydney and the Internal Research Review Committee of The Cancer Council approved this study.

## RESULTS

### Participants

In all, 30 of 53 eligible patients agreed to participate, with 26 completing an interview. Two patients became too ill and two were unavailable in the study period. There were 15 women and 11 men. Five participants were younger than 50 years, 15 were 50–69 years and six were 70 years or older. Half had more than 10 years of schooling and 13 were married or in a defacto relationship. Participants had been diagnosed with 11 different types of cancer, the largest single number of participants having breast cancer (seven). No more than three participants had any other single type of cancer. In all, 11 participants had been diagnosed within the past year and six more than 5 years ago. A total of 20 participants had undergone chemotherapy, 15 surgery and nine radiotherapy. Nine participants had ever used the Internet, most daily. The reasons for never having used the Internet were lack of access, knowledge or interest, being too old and laziness.

### Patient understanding of statistical information

Table 1

**Table 1:** Participants' understanding of statistical concepts

**Concept**	**Number of participants who understood the concept (*n*=26)**
Change to remaining lifespan	18
Absolute survival	16
Median survival	8
Graph	6
Conditional survival	2
Relative survival	1

shows the number of people who correctly interpreted each statistical concept. *Median survival* was commonly misinterpreted as the *average*, which participants felt was a more common and better-understood term. *Conditional* and *relative survival* were poorly understood, with participants applying the definition of absolute survival to these concepts.

### Content analysis

**(1) General support for written information about survival** A total of 23 participants supported the provision of written prognostic information outside the clinical context. Six of these participants expressed a prescriptive attitude towards this. Two participants said:

I don't suppose [all] people would like to see that [information] but they should. [Participant 24] Because people diagnosed with cancer…always should know what their chances are to give[them] the positive frame of mind. [Participant 13]

Eight of these participants were not personally interested in receiving this information. Six preferred to get it from their doctor and two were physically well and did not consider themselves cancer patients.

One participant felt that this information should be provided only to patients with a poor prognosis.

Two participants felt that written prognostic information should not be provided at all. One of them said:

This is generalised information, which is not really good. [Participant 15]

(a) *Meaning of prognostic information* Conveying hope was said to be a fundamental purpose of prognostic information. This view was evident in the strong preference for information expressed as survival and hope-preserving presentation styles. Even participants concerned about information clarity acknowledged the need for it to convey hope.

(b) *Reasons for providing prognostic information* Support for providing written prognostic information comprised three themes: (i) better information to assist decision-making; (ii) psychological and emotional benefits; and (iii) reduced patient burden by assisting them to explain their situation to significant others. The first category included the provision of accurate, reliable and accessible information; facilitating patient understanding by providing an indication of how long people live; facilitating question asking for further information gathering; and assisting decision-making. Two participants said:

Having the knowledge about your possible prognosis leads you to ask questions. [Participant 25] So for me I really needed to know [the chance of surviving] to know whether it was worth going through [treatment]. [Participant 2]

Some used this information as a coping mechanism. It gave them a sense of control, reduced anxiety and provided hope and reassurance by showing that people in a similar situation have a future. One patient said:

Because people worry themselves sick if they don't get [the information]. [They] imagine they're in a worse situation than they really are. [Participant 20]

(c) *Reasons against providing prognostic information*Participants nominated cognitive and emotional reasons against providing this information. Cognitive reasons focused on the inaccuracy and lack of specificity and meaning of statistics, and the potential for information to be misunderstood. Two participants commented:

I don't think [knowing the statistics] would change the end result and whether I live or not. [Participant 3] I mean if you put the information in a booklet that says, ‘if you get cancer, you can live for another five years or you can die within three years’, I don't think that sort of helps anybody. [Participant 4]

The emotional reasons included the negativity of the information and its potential to destroy hope and cause anxiety and depression. Two participants said:

Trouble is a lot of people read these things like it's a death sentence. [Participant 15] Yes I think it makes people anxious because people are frightened of dying or what's going to happen to them and they worry about it. [Participant 4]

**(2) Preferences for the format of information**

(a) *Framing*In all, 25 participants wanted positively framed information because the word *survive* and the open-ended timeframe communicated hope. Two participants said:

It's just the word survive. It's the one which gives you hope. [Participant 3] Yes it does [give hope]. Hopefully within that timeframe they could come up with something that could save you. [Participant 19]

One participant preferred the mixed frame because it enabled patients to choose what they would focus on, although he personally would focus on survival.

All participants who preferred positive framing were against expressing information as mortality because it evoked negative reactions including fear, anxiety and depression. Three participants commented:

Not encouraging. You look at the words and think I've got no chance. Morbid. [Participant 18] I don't think you should put down the word die. Die. You're all going to die. [Participant 16] You see the amount who die, it sounds like a death sentence. [Participant 8]

They felt that negative framing could be inferred from positive framing and so did not need to be highlighted. One participant said:

If you know that 50% will survive you don't need to be told that 50% will die. I mean what else is going to happen to them! [Participant 22]

(b) *Content*Generally, participants preferred positive information. For 12, this was a long timeframe regardless of the proportion who reached it. The fact that some people lived a long time gave them hope that they would too. One participant said:

The first thing to hit you is the number of years, that's the first thing you look at. You may not be able to understand the percentages but you zero in [on time], absolutely. [Participant 6]

For others, a high survival rate was positive. One patient commented:

Just the number, 60% will live at least five years, it's a fairly high rate, not as bad as 57%. [Participant 8]

Participants' content preferences were influenced by the meaningfulness and relevance of the information. *Relative survival* and *change to life expectancy* were the least preferred types of information (Table 2

**Table 2:** Participants' content preferences ranked in order of most to least selected

**Concept**	**Most preferred**	**Least preferred**
Conditional median	1	4
10-year absolute	2	7
5-year absolute	3	4
Conditional absolute	3	7
Conditional relative	3	4
Change to life expectancy	6	3
10-year relative	6	2
Median	6	9
5-year relative	9	1

). Participants felt that these statistics were an unfair comparison between cancer patients and healthy people and had no meaning as they only emphasised what they already knew, that they are more likely to die sooner than ‘*other people*’. Two participants commented:

I mean you've got cancer so you really don't care about what's happening to people who don't. [Participant 16] When you compare it to other women, you're not staying within the boundaries of just cancer patients. The information I'd like to see is to compare cancer patients alone… [Participant 26]

(c) *Presentation Emotional effect*: Graphs were consistently considered negative as they highlighted the ever-increasing mortality. Six participants interpreted the arbitrary cut-off point for time post-diagnosis as the longest anyone could live. Two participants said:

When they see the graph they get a negative attitude and think ‘that's it’. [Participant 7] Yes I would [be anxious]. I mean all of a sudden you're off the scale. [Participant 5]

Other presentation formats were considered more positive. However, the faces evoked intense negative responses in some participants for their superficiality and the vividness of the mortality information.

*Clarity*: With the exception of the graphs, participants were generally more concerned about the clarity of the information than the presentation format. One patient observed:

So long as it's clear it doesn't really matter how it's presented. [Participant 19]

They wanted information presented simply to facilitate understanding. This included using as few words as possible, combining presentation styles, for example, using faces and numbers together and using plain language instead of statistical terminology. Two participants said:

It spells the information out pretty succinctly, you don't have to sort through lots of words. [Participant 2] Personally layman's language is better. You've got to suit the masses. [Participant 16]

Nevertheless, participants felt that the faces and joint number–percentage presentation provided the clearest way of presenting prognostic information. In particular, participants who preferred the faces said the colour division between survival and mortality would be useful for people with low literacy levels or from non-English speaking backgrounds. One participant commented:

Two different colours means you can differentiate. Doesn't matter what it is, any colour at all. [Participant 8]

However, others preferred information presented as numbers only. They said that percentages were harder to interpret than numbers, which people are more familiar with and could more easily visualise. Two participants said:

What do percentages mean to people who don't deal with percentages. [They don't] mean much. [Participant 6] I'd say 60 out of 100, people relate to that more so than a percentage even though it's out of 100. [Participant 8]

*Information should be clear but not confronting*: Participants commented on the tension between the need for clear information and the desire for hope. Most wanted clear, not confronting, information. This tension was most evident in the shift away from faces as a preferred presentation style when mortality outweighed survival. In particular, participants said the faces made mortality too vivid and unnecessarily emphasised what would be clear from a sentence or number. They said the latter was no less clear, just less confronting. One patient said:

When you read it [as text] it doesn't seem so bad, but when it's the dots it's a bit more negative. [Participant 8]

(d) *Differentiation by stage* Nine participants wanted information on all stages as it gave them an idea of what to expect in the future. Six participants wanted information only for their stage, as information on other stages was not relevant because their cancer may never progress. They felt that such information would cause them to worry unnecessarily as it highlighted disease progression and the associated increase in mortality. One participant said:

I think because nobody wants to think about it once it spreads from one stage to [the next] and then from that to [the next].…I think it's more depressing. Without even knowing the figures it is depress[ing]. [Participant 3]

Four participants said their preference for one or three stages was affected by the difference in survival between the different stages. Where there was a large difference, they would prefer to see information for one stage only.

**(3) Perceived influences on the use of, and preferences for, written information**

(a) *Age* Age was thought to influence the information wanted, its emotional impact and how people accessed information. Five participants believed that older people would generally be less interested in and upset by prognostic information as they had already lived most of their life and would expect to die soon. One participant said:

It would possibly help younger people because they'd be more worried. I've got to the stage where I'm not worried about whether I'm going to die or not. I think I've had a pretty good life, I'm 82 years old. [Participant 4]

However, not all participants held this view. One participant commented:

The impact is still as great I suppose whether you're 20 or 120. [Participant 10]

Participant said booklets were more important than the Internet as older people would be less likely to have Internet access. One participant said:

It's mostly older people who've got this condition. They probably won't have computers. [Participant 6]

(b) *Time since diagnosis*:Four participants suggested that time since diagnosis affected the relevance of information based on different time periods. For example, information about 10-year absolute survival would be more relevant for someone 5 years postdiagnosis than someone recently diagnosed. Two participants said:

It does depend though where you are because say 12 months down the track you might be looking for information [about now] I've got this far, what happens next. [Partcipant 9]

There isn't much point looking ten years down the track. [Partcipant 9]

In addition, participants felt that those further postdiagnosis would be better adjusted to their diagnosis and planning a future, and therefore place more importance on information clarity than hope.

Participants who said time since diagnosis had no influence, acknowledged that information would only be relevant if it pertained to a time period not yet reached. For example, 1-year absolute survival would only be relevant for people who had been diagnosed for less than 1 year.

(c) *Emotional coping*Emotional coping was seen to both influence and be affected by the framing, content, presentation and differentiation by stage of prognostic information. For instance, framing information as mortality could result in anxiety, depression and a ‘*why bother, I'm going to die anyway*’ attitude. In the same way, people's general attitude towards life and their ability to cope emotionally with their illness was thought to impact on their preferences. For example, people with a more positive outlook on life might be less affected by mortality information than someone with a negative outlook. Two participants said:

It depends how you're feeling at the time. You're feeling totally vulnerable or you've got past that stage and you're feeling like ‘hey I'm going to beat this, just give me the facts’ or if you want anything softer. [Participant 2] I think [the emotional impact] depends on the type of person. If they're defeatist in their attitude to anything in life then they're going to look at those figures and say well gee I don't like my chances. [Participant 3]

(d) *Credibility of information source*In all, 12 participants felt that written prognostic information should include details about the source of The Cancer Council's statistics, The New South Wales Central Cancer Registry. Seven participants felt that the credibility of The Cancer Council implied any information appearing in their materials or on their website, regardless of source, was accurate and reliable. A third group of participants saw this issue as irrelevant. They had previously experienced difficulty in finding cancer-related information and would take whatever they could find regardless of source.

Eight participants said they would check the date of the information. Generally they expected annual update of Internet information and biennial of booklets.

### Acceptability and utility of a card sort for eliciting understanding and preferences

Overall, participants said that the card sort method was acceptable and made it easy to pick their interpretation of a statistical concept. Three participants had difficulty understanding what was asked of them in relation to interpreting the relevant statistical concept and only one said that the card sort felt like a test.

For preferences, participants said that the ability to pick up and move the cards facilitated comparison between cards, making it easier to choose preferences. However, a minority found nine content choices ‘*too much*’ and the presentation of each content area using each 5- and 10-year timeframes repetitive and confusing. These participants tended to group statistics into types of information and then pick their preferences from their most and least preferred groups of information. Thus it appears that at least for some participants, content preference was based on a combination of the type of information, timeframe and probability of survival.

Four participants had problems with the use of women aged 75–89 years as the exemplar group, particularly with respect to discussing the emotional impact of information, which they perceived to be minimal given ‘*people that age would expect to die soon and [would be] less upset by [prognostic information] than a younger person*.’ [Participant 18] These participants said that it was easier to comment on the emotional effect of information when they applied it to someone their own age.

## DISCUSSION

Overall, most participants felt that positive, clear and relevant written prognostic information should be made available to patients outside the clinical context. In addition to these issues, the study has shown that the card sort used, although not without problems, is an acceptable way of eliciting patients' understanding of, and preferences for, statistical information about survival.

### Patient's understanding of statistical information

An Australian study involving women with stage I and II breast cancer found that 73% of women misunderstood median, often interpreting it as the average. Lack of understanding among participants in this and the current study suggests that care needs to be taken when using statistics in prognostic information, regardless of setting. Most participants said that they understood concepts better when the appropriate statistical terminology was replaced by plain language. This may be useful for improving, but does not guarantee, patient's understanding.

### General views about prognostic information

The strong preference for prognostic information in this study is consistent with a recent large-scale British study (Jenkins *et al*, 2001), which found that 75% of patients want to know their chance of cure. Participants' reasons for and against providing written prognostic information independent of the clinical context are similar to doctors' concerns regarding the provision of information in the clinical context (Oken, 1961; Christakis, 1999). In both situations, the focus is on the potential for psychological benefits and harm. These similarities suggest that there may be no difference in the potential patient effects attributed to verbal information in a clinical context *vs* written information independent of the doctor.

### Preferences for written prognostic information

The strong preference for positively framed information in this study is inconsistent with a previous study on framing preferences (Lobb *et al*, 1999). Unlike the current study, which was context free, previous research has assessed framing preferences in the context of choosing a treatment. This suggests that people prefer different frames in different contexts. This possibility merits investigation.

### Influences on preferences

Participants' said age, time since diagnosis, emotional coping and source credibility would affect preferences for, and the use of, written prognostic information. The belief that older people would be less interested in prognostic information is consistent with a previous finding that people older than 70 years of age are less interested in prognostic information than younger people (Jenkins *et al*, 2001). Although this contradicts earlier work (Meredith *et al*, 1996), it provides a possible explanation for physicians' greater willingness to provide a frank prognosis to older patients (Lamont *et al*, 2001).

It is important to note that no firm conclusions can be drawn from this study about the influence of age, time since diagnosis, emotional coping and source credibility on preferences for and the use and emotional impact of this information. A large representative sample of cancer patients would be required to assess whether these suggested influences have an effect in practice.

### Limitations

Given the qualitative design, convenience sample and small sample size, the results of this study cannot be generalised with any great certainty. A larger quantitative survey of a representative sample of cancer patients would be necessary to determine the prevalence of the preferences and perceptions found in this study and to give greater certainty to their relative importance.

A few participants had problems with the card sort, particularly the age group chosen for the example and the number of cards. Future users of this method should aim to use fewer cards than the most we used (nine). A single age group was necessary to keep the information to a manageable level. It would, however, be possible to match example age groups to participants' ages and this could be a preferred approach for future use.

## CONCLUSION

We believe this to be the first study to explore whether cancer patients want *written* prognostic information in the form of booklets and the Internet, independent of their doctor; their preferences for the framing, content, presentation and differentiation by stage of information in this context; and the reasons underlying these preferences. Despite the limitations of this study, it addresses criticism that consumers are rarely included in developing consumer information materials (Coulter *et al*, 1999). In addition, the study has identified two fundamental and sometimes conflicting factors underlying patient preferences: communication of hope *vs* understanding, and influences of particular patient characteristics on preferences. These factors need to be taken into account when designing written prognostic information for cancer patients.
